# Nakalanga Syndrome: Clinical Characteristics, Potential Causes, and Its Relationship with Recently Described Nodding Syndrome

**DOI:** 10.1371/journal.pntd.0005201

**Published:** 2017-02-09

**Authors:** Kathrin Föger, Gina Gora-Stahlberg, James Sejvar, Emilio Ovuga, Louise Jilek-Aall, Erich Schmutzhard, Christoph Kaiser, Andrea S. Winkler

**Affiliations:** 1 Department of Neurology, Technical University of Munich, Munich, Germany; 2 Division of High-Consequence Pathogens and Pathology, National Center for Emerging and Zoonotic Infectious Diseases, Centers for Disease Control and Prevention, Atlanta, Georgia, United States of America; 3 Department of Psychiatry and Mental Health, Gulu University, Gulu, Uganda; 4 Department of Psychiatry, University of British Columbia, Vancouver, Canada; 5 Department of Neurology, Medical University Innsbruck, Innsbruck, Austria; 6 Pediatric Practice, Baden-Baden, Germany; 7 Centre for Global Health, Institute of Health and Society, University of Oslo, Oslo, Norway; Federal University of Agriculture, NIGERIA

## Abstract

Nakalanga syndrome is a condition that was described in Uganda and various other African countries decades ago. Its features include growth retardation, physical deformities, endocrine dysfunction, mental impairment, and epilepsy, amongst others. Its cause remains obscure. Nodding syndrome is a neurological disorder with some features in common with Nakalanga syndrome, which has been described mainly in Uganda, South Sudan, and Tanzania. It has been considered an encephalopathy affecting children who, besides head nodding attacks, can also present with stunted growth, delayed puberty, and mental impairment, amongst other symptoms. Despite active research over the last years on the pathogenesis of Nodding syndrome, to date, no convincing single cause of Nodding syndrome has been reported. In this review, by means of a thorough literature search, we compare features of both disorders. We conclude that Nakalanga and Nodding syndromes are closely related and may represent the same condition. Our findings may provide new directions in research on the cause underlying this neurological disorder.

## The Terms of “Nakalanga” and “Nodding Syndrome”

The term Nakalanga is first found in an anthropological account published by John Roscoe [1] in 1911, mentioning it as the name of a god recognized by a clan of the Buganda kingdom living in the area of the Mabira forest located 60 km east of today`s Ugandan capital city, Kampala. Up to today, Nakalanga is still the name of a god in the conceptual world of the Baganda [2]. In addition to this meaning, in a 1934 geographical article about the Mabira forest, the word is found used to designate a person characterized by a condition of short stature (“dwarfism”), which was frequently seen in the area [3]. It was suggested that these persons were descendants of a pygmy tribe that in bygone times would have inhabited the Mabira forest [3,4].

**Fig 1:**
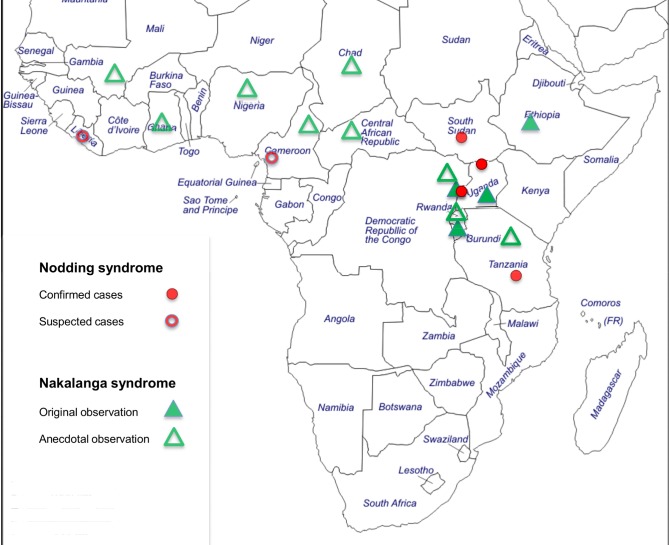
Map of sub-Saharan Africa. Cases of Nakalanga and Nodding syndrome are reported from onchocerciasis endemic areas throughout West, Central, and East Africa.

During the late 1940s, Raper and Ladkin [5] carried out an extensive investigation in the Mabira forest on patients affected by the Nakalanga phenomenon. They gave a thorough description of the condition and concluded that their patients were affected by a medical disorder, which they called Nakalanga syndrome. Up to 1965, two additional case series were published from the same area [6,7]. Thereafter, no more studies were undertaken, and when the Mabira forest became accessible again in the 1980s, no more cases were found. Instead, patients with a condition conforming with Nakalanga syndrome were observed in other locations, namely western Uganda [8–11], Burundi [12], and Ethiopia [13]. This is complemented by anecdotal observation from other areas of sub-Saharan Africa [14–16], suggesting that the syndrome is probably not confined to Mabira and southeastern Uganda (Fig 1). As early as 1938, from an area in Mexico where a high endemicity of onchocerciasis and an elevated epilepsy prevalence were found, Casis Sacre described patients affected at a young age by a severe disorder characterized by growth failure, mental retardation, generalized weakness, and disturbed pubertal development [17]. Although the similarity of this observation with the above-mentioned reports was recognized [18], no further investigations from this area are available to more precisely assess its possible connection with Nakalanga syndrome.

Nodding syndrome is an unexplained neurological disorder with the clinical core feature of a paroxysmal spell in which the head repeatedly bobs forward for several minutes or longer [19]. It emerged during the last two decades in then southern Sudan and northern Uganda [20–26] and, in retrospect, was documented in Tanzania since the 1960s [27,28] and in western Uganda in 1994 [29]. Reports from Grand Bassa County, Liberia [30], from the Littoral Province of Cameroon [31], and from the Ituri District in the Orientale Province of the Democratic Republic of the Congo [32] also point towards the possible existence of Nodding syndrome in these areas (Fig 1). A case definition for Nodding syndrome was agreed upon at an international conference in Kampala, Uganda in 2012 [33]. Because patients with Nodding syndrome frequently display signs and symptoms that have also been described in Nakalanga syndrome, it has been questioned if both these conditions represent different manifestations of a common underlying pathology [21,22,34–37].

In this article, we focus on the following aspects:

- Based on a thorough literature search, we summarize the characteristic clinical features of Nakalanga syndrome as they were originally described in different areas, and we propose a working medical definition.
- We summarize available information on the etiology and possible causation of Nakalanga syndrome.
- We examine the question of whether the symptoms and signs found in Nakalanga syndrome are also present in cases of Nodding syndrome.
- We give suggestions as to how the relationship between the two entities could be further studied.

## Characterization of Nakalanga Syndrome and a Proposed Definition

We searched several medical databases (Medline, ScienceDirect, African Neurology Database of the Institute of Neuroepidemiology of the University of Limoges) using the search term “nakalanga.” Other sources, such as commercial search engines or unpublished congress proceedings, were searched without specific limits, and reference lists of retrieved articles and reviews were screened for further records of relevance (latest search: May 23, 2016). The database search identified 45 records, and 6 additional records were found from other sources. After removal of duplicate entries and assessment of relevance, 12 articles were found to present original clinical data [5–14,38,39]. Three of these were excluded because they contained redundant or insufficient information [11,38,39]. The remaining nine records [5–10,12–14] represent the evidence base for a systematic characterization of Nakalanga syndrome (Table 1). A flow diagram describing the search procedure is available as supporting information (S1 Diagram).

**Table 1:** Clinical case series and case reports on Nakalanga syndrome: Southeast Uganda 1950–64, Ethiopia 1967, West Uganda 1991–95, Burundi 1995.

Publication [Reference]	Age	Anthropometry		Clinical Features					Onchocerciasis^ii^
	at onset^i^/ examination	Stunting^i^	Wasting^i^	Pubertal Development	Mental Development	Facial Dysmorphia	Kyphosis Scoliosis	Epileptic Seizures	
**Southeast Uganda 1950–64**									
Raper and Ladkin [5]	n.r.^iii^ (2–4)/ 11.5 (6–18)	-4.54^iv^ (-1.52–-6.15)	-6.21^iv^ (-3.12–-9.84)	delay; small genitalia; some cases retarded ^v^	not severely impaired; appearance younger^v^	small mandible; protuberant skull; large lips^v^	prone to develop scoliosis^v^	Epilepsy in 2/7	Positive in all cases
Marshall and Cherry [14]^vi^	n.r.^iii^/ 30	-5.06 ^iv^	n.r.^iii^	few pubic hair; normal testes, small penis	mentally dull	prognathic jaw; protruding teeth	n.r.^iii^	no epilepsy	positive
Jeliffe et al. [6]	n.r.^iii^/ 14 (10–19)	-4.80 ^iv^ (-1.08–-5.58)	-3.53 ^iv^ (-1.46–-6.54)	reduced sexual activity; poor pubic hair^v^	Mild to moderate apathy; 2/ 5 “mental torpor”	prognatic jaw, forward angulated incisors in 2/ 5	2/5 marked kyphosis	epilepsy in 1/5	Positive in all
Bagenda et al. [7]	n. r. (0–7)/ 20 (13–25)	-4.72 ^iv^ (-1.98–-8.52)	n. r.^iii^	delay; cases ≥20 y in puberty or postpuberty	7/ 19 normal life; 10 doing simple jobs; 2 looked after	deep-set eyes; teeth protruding; small chin^v^	8/19 kyphoscoliosis	epilepsy in 7/19	Positive in 18/19
**Ethiopia 1967**									
Oomen [13] ^vi^	n.r.^iii^/ 35	-5.74^iv^	cachectic^v^	small genitalia	n.r.^iii^	n.r.^iii^	kyphoscoliosis	epilepsy	positive
**West Uganda 1993–94**									
Ovuga et al. [8]^vii^	n.r.^iii^	growth arrest^viii^	emaciation^v^^,^^viii^	poorly developed^v^	31/91 cognitive impairment; some apathetic^v^	distorted dentition^v^	pigeon chest; kyphosis^v^	epilepsy in 24/91	Positive in 82/91
Kipp et al. [9]^vii^	7 (1–>8)/ 18 (15–32)	-4.9^ix^	-1.9^ix^	27 of 31 prepubertal, versus 2 of 28 controls	several cases mentally subnormal^v^	n.r.^iii^	deformed spine in 8/31	n. r.^iii^	Positive in all
Höfer [10] ^vii^	n.r.^iii^ (1–11)/ 15 (7–20)	-4.02 ^iv^ (-1.36–-6.62)	-2.53^iv^ (+0.51–-10.7)	delay, in comparison with local controls	14/36 mentally retarded	6/36 protruding forehead, flat nasal bridge	Kyphoscoliosis in 6/36	Epilepsy in 16/36	Positive in 13/16
**Burundi 1995**									
Newell et al. [12]	n.r.^iii^ / 17 (12–20)	-4.83^iv^ (-2.59–-5.19)	-1.11^iv^ (+1.18–3.64)	delay in 2/9	n.r.^iii^	n.r.^iii^	n.r.^iii^	all with epilepsy	Positive in all

^i^ median (range)

^ii^ detection of microfilaria of *Onchocerca volvulus* in skin biopsy, or of antibodies in serology (Newell et al. [12])

^iii^ n.r. = not reported

^iv^ z-score for height-for-age (stunting) and body mass index (BMI) (waisting), referring to 2006 WHO growth standard [40,41]. http://www.who.int/childgrowth/en/

^v^ no quantified information

^vi^ case report

^vii^ overlap of study populations in studies of Ovuga et al. [8], Kipp et al. [9], and Höfer [10]

^viii^ growth arrest as mandatory symptom, defined as <80% of average height of local population; emaciation as characteristic symptom, not specified

^ix^ mean z-score of height-for-age and BMI, referring to 1977 NCHS growth reference [62]. http://www.cdc.gov/nchs/data/series/sr_11/sr11_165.pdf; Range not reported

In their pivotal study in the Mabira forest, Raper and Ladkin [5] collected information from various community members about the local concept of the Nakalanga phenomenon. As a second step, in a convenience sample of characteristic patients, a list of signs and symptoms was established, and this was completed by more detailed investigations in seven selected patients, who were taken to a nearby hospital. Similar studies were carried out by Jellife et al. [6] and Bagenda et al. [7] in areas neighboring Mabira forest.

As an overall result of these studies, Nakalanga syndrome was specified as a disorder affecting previously healthy children during the first ten years of their life. Growth retardation was described as the most prominent feature. When available, we applied height data from the original publications [5–7,14] to the currently used WHO growth standards [40–42] and found strikingly similar median z-scores between -4.54 and -4.80 in the case series from these three closely neighboring areas [5–7], indicating an overall severe degree of stunting (Table 1). Generally, a height-for-age z-score of more than 2.0 standard deviations below the growth standard median is considered to indicate stunted growth [40]. Yet, in each of the three studies [5–7], one patient with a height measurement corresponding to a z-score above the -2.0 standard deviation threshold was found (z-scores: -1.08, -1.52, and -1.98; Table 1), indicating a normal stature even when related to the current WHO standard. This is evidence that stunted growth, although reported as a frequent symptom, was not a mandatory criterion in the concept of the local communities to classify a person as a Nakalanga patient. The height of 53 local control children was measured by Raper and Ladkin [5] and, expectedly, was found at z-scores below the current WHO standard (median: -2.14; range: +1.4 to -5.3). Twelve of these 53 children had height-for-age z-scores below -3.0, but their overall height was still taller than that of Nakalanga patients. Emaciation, delayed sexual development, and mental impairment were described as additional characteristic symptoms of Nakalanga syndrome, although these were not invariably found in all cases. Some patients also showed facial dysmorphia, deformation of the vertebral spine, or epileptic seizures (Table 1).

In 1992, Ovuga et al. [8] for the first time described the unusually frequent occurrence of a condition similar to Nakalanga syndrome in the Itwara onchocerciasis focus, situated in the Kabarole district, western Uganda (Table 1). The Itwara forest has no contiguity to Mabira forest in southeastern Uganda, where Raper and Ladkin had made their primary investigations 40 years earlier [5]. The study of Kipp et al. [9] demonstrated that “Ekihuruka,” as the disorder was named by the population in the local Rutoro language, was consistent in its clinical picture with Nakalanga syndrome (Table 1). In 1999, a detailed analysis of 36 patients with Nakalanga (Ekihuruka) syndrome examined in the mentioned area of the Kabarole district was reported [10]. A subgroup of 12 of these patients, who in addition were suffering from epilepsy, were also followed in the study of Kaiser et al. [11,43]. In accordance with the earlier reports from southeastern Uganda, the study of Höfer [10] demonstrated that patients affected by Nakalanga (Ekihuruka) syndrome were not solely characterized by growth retardation or another single sign or symptom alone but rather by the combination of several distinct signs and symptoms. Consistent with the anthropometric results from southeastern Uganda [5], the height measured in a control group of healthy children in western Uganda overlapped with the upper range of Nakalanga (Ekihuruka) patients [10], although the height-for-age z-score of the control group (median: -1.09; range: +0.96 to -2.8) was by far higher than that of the patient group (median: -4.02; range: -1.36 to -6.62; Table 1).

In 1997, Newell et al. [12] in a study from an onchocerciasis endemic area in Burundi reported cases with retarded growth, mental impairment, and delayed sexual development among a group of patients identified in an epilepsy survey (Table 1), and they pointed out the similarity of this observation with Nakalanga syndrome. In a study on clinical features of onchocerciasis in Ethiopia, Oomen described an individual patient showing a striking similarity with Nakalanga syndrome as found in southeastern Uganda [13].

The consistent syndromic pattern found in the mentioned noncontingent areas indicates that the reported patients may be affected by the same neurological disorder. Based on these findings, we propose that Nakalanga syndrome can be defined as a disorder affecting young children and youths who were born healthy and have passed through an initial phase of healthy development (Table 2). Patients present with at least one of the following predominant symptoms: retardation of growth (stunting), emaciation (wasting), delayed sexual development, or mental impairment. Less frequently, facial dysmorphia with small mandible, large lips, protruding front teeth, kyphoscoliosis, or epileptic seizures are encountered along with the mentioned major signs and symptoms. In the environment of a patient with Nakalanga syndrome, an accumulation of other cases with similar features is found.

**Table 2:** Proposed criteria for definition of Nakalanga syndrome compared to criteria for definition of a probable case of Nodding syndrome consented in Kampala 2012 (adapted from ref. [26,33]).

Proposed criteria for Nakalanga syndrome	Consented criteria for Nodding syndrome
**Major criteria (obligatory)**	**Major criteria**
1. Born healthy	1. Reported head nodding in a previously healthy person
2. Onset in childhood	2. Age 3–18 y at onset of head nodding
3. No alternative explanatory condition	3. Nodding frequency 5–20 times/min
4. Plus, one or several symptoms of developmental disturbance:	
a) Stunting, growth failure	
b) Wasting, emaciation	
c) Retardation of sexual development	
d) Mental impairment	
**Minor criteria (additional)**	**Minor criteria**
5. Facial dysmorphia: small and protuberant mandible, large lips, protruding front teeth	4. Other neurological abnormalities (cognitive, school dropout, other seizures, or neurological abnormalities)
6. Kyphoscoliosis	5. Clustering in space or time with similar cases
7. Epileptic seizures	6. Delayed sexual or physical development
8. Occurrence of similar cases in the surroundings	7. Psychiatric manifestations

## Information on Etiology and Possible Causes of Nakalanga Syndrome

In view of the clinical picture and the epidemiologic distribution, Raper and Ladkin [5] excluded the hypothesis that people with Nakalanga syndrome could belong to a pygmy tribe. The combination of their clinical observations with the finding of a low urinary excretion of the 17-ketosteroids led them to assume that Nakalanga syndrome should be induced by defects in pituitary and adrenal gland function. In 1961, Marshall and Cherry [14] reported on an autopsy case which revealed no major changes of the histological appearance of the adrenal gland compatible with major adrenal dysfunction. In the histology of the pituitary gland of this case, they described some alterations in the composition of acidophil, basophil, and chromophobe cells, but the vascular supply was not disturbed, and there was no major lesion found that could have been produced by invasion of a pathogenic agent, e. g., *Onchocerca volvulus* microfilaria. It was concluded that “the primary affection may have been either in the pituitary portal system or the hypothalamus,” and the authors speculated that the “humoral transmission through the pituitary portal system was possibly jeopardized by circulating products of dead microfilaria” [14]. In a series of ten Nakalanga patients, Leonard and Stanfield [39] found normal levels of morning plasma cortisol and a normal protein binding fraction of cortisol. They also reported normal results of fasting serum growth hormone (GH) in two patients and a normal rise of GH in a third case following stimulation with injected insulin [39]. In the studies from Burundi [12] and from western Uganda [10], thyroid-stimulating hormone (TSH) as well as total thyroxine (T4) and triiodothyronine (T3) levels were measured and normal values were found in 31 of 32 cases. Höfer [10] also investigated the serum concentration of insulin-like growth factor I (IGF-I) and IGF-binding-protein-3 (IGF-BP-3) in a group of 23 severely stunted Nakalanga patients and found slightly lower concentrations in patients than controls, but IGF-I levels were not compatible with GH deficiency in 16 cases. When the remaining seven patients were assessed case-by-case with GH determination after physical strain and a follow-up of their growth rate over one year, GH deficiency was excluded in three more patients. In agreement with the findings of Leonard and Stanfield [39], and contrasting with the earlier assumptions of Raper and Ladkin [5], the results of Höfer [10] disprove the hypothesis that a primary pituitary lesion plays a causative role in the pathophysiology of Nakalanga syndrome.

When Nakalanga syndrome was first recognized in the 1950s as a regional medical entity [5–7], a search on the cause of the condition was undertaken with the resources available at the time. On the ground of their clinical, laboratory, and epidemiological findings, the authors of the three studies from Southeast Uganda widely excluded a number of chronic disorders that elsewhere in Uganda or tropical Africa had been found related with growth failure [5–7]. Explicitly, they found no connection with malaria, malnutrition, tuberculosis, rickets, syphilis, blood dyscrasias, intestinal parasites, and chronic diseases of the liver or other organs, but viral etiologies or auto-immune disorders were not mentioned. In view of the sporadic distribution of patients in different ethnic groups and families, a genetic disorder was considered unlikely, although this was not fully excluded [6]. Consistent with the findings from southeastern Uganda, no known disorder could be identified which possibly caused growth failure in Nakalanga (Ekihuruka) patients in western Uganda [9,10]. As a consistent observation, all study areas were found highly infested with onchocerciasis (Table 1).

Little is known about the natural history and the prognosis of Nakalanga syndrome. According to Raper and Ladkin [5], after the onset in early childhood, the symptoms progress to reach a peak during adolescence. In some patients, the physical and mental condition visibly deteriorates to the extent of severe wasting and progressive loss of mental function. It seems likely that Nakalanga syndrome results in increased mortality, and this is supported by the observation that few, if any, Nakalanga patients older than 30 years were reported in the various studies (Table 1). In the cohort of 12 patients in western Uganda, who in addition to Nakalanga (Ekihuruka) syndrome were also suffering from epilepsy, a high mortality was found [11]. No comparable data have been assessed from Nakalanga patients without associated epilepsy. Contrasting with the observation of an obviously severe course of the disease in many patients, it has also been stated that some Nakalanga patients “may reach an advanced age” [5] or “appear to live a normal life” [6].

### Comparison between Nakalanga Syndrome and Nodding Syndrome

A second literature search for records presenting original clinical information on patients with Nodding syndrome retrieved 20 publications [19–25,28,29,44–54]. Nine of these were excluded from the analysis [19,22,24,25,46–48,51,52] because they presented incomplete data or redundant information, which was found in more detail in another article. The remaining 11 articles were systematically screened for symptoms and signs, which beforehand had been identified as characteristic for Nakalanga syndrome (Table 3). Details of the search procedure and a flow diagram is available as supporting information (S2 Diagram).

**Table 3:** Clinical features of Nakalanga syndrome according to the proposed definition found in patients with Nodding syndrome.

Publication [Reference]	Age^i^	Clinical Features						
	At onset of NS / at examination	Stunting	Wasting	Delayed Puberty	Mental Retardation	Facial Dysmorphia	Kyphosis Scoliosis	Epileptic seizures other than head nodding
**South Sudan**								
Lacey [20] ^ii^	n. r.^iii^ / 18	+^iv^^,^^v^	+ ^iv^^,^^v^	n. r. ^iii^	+^iv^	n. r. ^iii^	n. r. ^iii^	**+**^iv^
Nyungera et al. [45]	n. r.^iii^ / 15	+ ^iv^^,^^v^	+ ^iv^^,^^v^	n. r. ^iii^	+^iv^	n. r. ^iii^	n. r. ^iii^	**+**^iv^
Tumwine et al. [21]	12 / 12	+ ^iv^^,^^v^	+ ^iv^^,^^v^	+^4^	+^iv^	n. r. ^iii^	n. r. ^iii^	**+**^iv^
De Polo et al. [53]	9 / 12	n. r. ^iii^	n. r. ^iii^	n. r.	all, different degree	n. r. ^iii^	n. r. ^iii^	all
**Northern Uganda**								
Idro et al. [24]	6 / 14	9/22 cases^v^	16/22 cases^v^	n. r. ^iii^	10/22 severely impaired	5/22 unspecified lip changes	1/22 kyphosis	18/22 cases
Sejvar et al. [23]	8 / 12	n. r. ^iii^	n. r. ^iii^	n. r. ^iii^	test score of cases lower than controls^vi^	n. r. ^iii^	n. r. ^iii^	6/23 cases
Kitara et al.[49]	11 / 13	n. r. ^iii^	**+**^v^	n. r. ^iii^	school failure	n. r. ^iii^	n. r. ^iii^	**+**^iv^
Piloya-Were et al. [50]	7 / 15	-2.41^vii^ (-0.10–-4.04)	n. r. ^iii^	obvious delay^viii^	n. r. ^iii^	n. r. ^iii^	n. r. ^iii^	5/8 cases
**Southern Tanzania**								
Winkler et al. [44]	n.r. ^iii^ / 14	n. r. ^iii^	n. r. ^iii^	n. r. ^iii^	12/62 impaired	n. r. ^iii^	n. r. ^iii^	34/62 cases
Spencer et al. [28]	10 / 13	11/33 with small stature	18/33 poorly nourished	n. r. ^iii^	8/33 impaired	n. r. ^iii^	n. r. ^iii^	29/33 cases
**Western Uganda**								
Kaiser et al. [29]^ii^	7 / 15	z-score^ix^ -7.3	n. r. ^iii^	infantile at age of 15 years	severely impaired	n. r. ^iii^	not present	present

^i^ median age in years

^ii^ case report

^iii^ n. r. = not reported

^iv^ **+** = feature reported without more specific information

^v^ no anthropometrical data reported

^vi^ clinical neurological assessment in 23 cases; Neurocognitive evaluation in 65 pairs of children and controls

^vii^ median (range) of z-score for height-for-age (stunting) referring to 2000 CDC growth standard [61]. http://www.cdc.gov/growthcharts/cdc_charts.htm

^viii^ Tanner maturity stage (TS) for breast or testis development in eight patients aged 13 to 18 years: TS1 in two patients (infantile), TS2 in five, TS3 in one

^ix^ z-score for height-for-age (stunting) referring to 1977 NCHS growth standard [62]. http://www.cdc.gov/nchs/data/series/sr_11/sr11_165.pdf

The age of Nodding syndrome patients at examination in cross-sectional studies, as well as the reported age at onset of the disorder, coincided well with that of patients affected by Nakalanga syndrome. Growth retardation and wasting as the most prominent features of Nakalanga syndrome were reported in the majority of published studies on Nodding syndrome, although exact measurements were only provided in two studies [29,50]. All studies on the clinical characteristics of Nodding syndrome also found mental impairment of varying degrees in a part of their patients, and all found patients with other epileptic seizures in addition to head nodding seizures. Disturbance of pubertal development, spine deformity, or facial dysmorphia were documented only exceptionally in patients with Nodding syndrome, but these features were not systematically investigated in all studies. The findings of Table 3 document the intriguing overlap between Nakalanga and Nodding syndromes, but hitherto available data are fragmentary and, in part, were established with poorly defined methods.

A recent study from northern Uganda [50] determined various endocrinological parameters in a series of eight patients with Nodding syndrome with different degrees of stunted growth and delayed puberty. Normal values were found for morning cortisol, TSH, and T4 in all patients. IGF-1 levels were generally low but within the normal range in five of eight patients, and serum GH—determined without stimulation—was normal. Gonadotropin levels were within the range of the clinically delayed stages of puberty. These results are consistent with those from patients with Nakalanga syndrome examined in southeastern Uganda [39], western Uganda [10], and Burundi [12]. As the authors state, pituitary stimulation tests are needed to better differentiate the role of hypothalamic and pituitary factors in inducing growth failure and hypogonadism in Nodding syndrome patients [50].

Our working definition of the Nakalanga syndrome shows an intriguing similarity to that of Nodding syndrome [33]: both conditions have their onset from an early age, are frequently combined with mental retardation, and there is a striking overlap in the symptom of growth failure as the predominant feature in Nakalanga patients. The patient with Nodding syndrome reported by Kaiser et al. [29] was also found severely stunted and considered by the community to suffer from Nakalanga syndrome, locally known as “Ekihuruka” [9,10]. This case report illustrates that Nakalanga and Nodding syndromes occur concomitantly in this area in western Uganda [8–11,29]. Possibly, some of the patients with Nakalanga syndrome reported 40 years earlier from southeastern Uganda were also affected by head nodding attacks. This is indicated by the description of Raper and Ladkin [5] that “inability to hold up the head is taken as a sign of a severe affection” and “certain cases would let the head fall forwards while eating and be quite incapable of raising it again.”

Another shared trait between Nodding and Nakalanga syndrome is the association with onchocerciasis, although this does not necessarily imply causality. All study areas where cases of either condition were reported were also highly endemic for onchocerciasis, and, when this was investigated, infection with *O*. *volvulus* was consistently more frequent in patients than in controls [9,10,21,22,25,46]. The isolated observation of a disorder reminiscent of Nakalanga syndrome from an onchocerciasis endemic area in Mexico could also be seen as corroboration of this association [17]. A close relationship between onchocerciasis and convulsive epilepsy has also been found in areas with and without confirmed Nodding syndrome [34,55], and there is indication that in these areas intensity of infection is involved in the induction of epilepsy [16,34,56]. So far, the issue of *O*. *volvulus* infection intensity has not been specifically examined in studies on Nodding or Nakalanga syndrome, but Raper and Ladkin pointed out their impression that Nakalanga patients “were more early and more heavily parasitised than their fellows” [5].

As a limitation to the assumption of a possible causative relationship between Nakalanga syndrome/Nodding syndrome and onchocerciasis, it is highly interesting that in many *O*. *volvulus* endemic areas, a single case of one of these two disorders was never reported. It has also not been clarified whether infection with *O*. *volvulus* actually precedes the onset of head nodding or growth retardation, because the relationship between onchocerciasis was so far investigated solely with cross-sectional studies. It may well be that Nodding and/or Nakalanga syndrome are due to an alternative pathogenic agent, potentially transferred by the same vector as *O*. *volvulus*. Affected children could be particularly susceptible to infection with onchocerciasis, which would then be a natural consequence rather than the cause of these disorders. As far as possible pathological mechanisms were investigated, the presence of the parasite, or evidence on an inflammatory or immune reaction to the parasite in the central nervous system, could not be verified neither in patients with Nodding syndrome [19,21,23,24,57,58] nor in the mentioned autopsy case affected by Nakalanga syndrome [14].

## Conclusions and Recommendations

Many aspects of Nakalanga and Nodding syndromes and their possible interrelation remain unsolved. One important question that needs to be addressed is the determination of the hitherto unknown frequency and the extent of growth failure in patients with Nodding syndrome in those areas where it was first confirmed—South Sudan, northern Uganda, and Tanzania. It is also unclear why in these areas no patients presenting with growth failure, delayed puberty, and mental retardation but without head nodding were reported, as this was demonstrated in southeastern as well as in western Uganda. We suggest that the characteristic features of Nakalanga syndrome should be more systematically investigated in patients with Nodding syndrome. It would also be of interest to find out whether the communities affected by Nodding syndrome also know about an illness in their area which is primarily characterized by growth failure and which might correspond to Nakalanga syndrome. We consider it of importance that studies on this issue uniformly refer to the generally consented 2012 Kampala case definition of Nodding syndrome [33,59] and that the effective ILAE guidelines for epidemiologic studies are followed [60]. Although sophisticated diagnostic tools such as video monitoring including electroencephalography (EEG) or brain imaging are not readily available in the setting of rural sub-Saharan Africa, seizures could be documented with mobile phone video and mobile EEG machines. These tools would be useful to confirm the diagnosis of Nodding syndrome in suspected cases. We want to encourage more neurological researchers to seek the collaboration of interested local colleagues to establish a dataset for neurophysiological records that could be reevaluated remotely by African and non-African experts alike.

Our proposed definition of the Nakalanga syndrome deliberately does not formulate specific criteria for confirmation of the disorder in the individual patient. We hope that our proposal will further develop on the basis of future research, and the contributions of others will help to improve its practicality. At present, we consider it of importance that the major features configuring Nakalanga syndrome—stunting, wasting, delayed sexual development, and mental impairment—should be assessed with clearly defined methodological approaches. Anthropometric measurements of height and weight should be carried out in patients, and in local controls, and should be related to the current standards of WHO [40–42]. These were developed with recent multicenter surveys and are recommended to replace earlier reference databases [61,62]. Definite clinical methods for the assessment of pubertal stages were developed with the basic studies of Marshall and Tanner [63,64], and these have been successfully applied in northern Uganda [50,65]. Because of the great diversity of regulating influences on the development of puberty [66–70], universal standards on the age at onset and the chronological sequence of pubertal stages are not available. It would therefore be crucial to always include carefully matched local controls in study protocols. The development and validation of more diagnostic tools on mental function would help to generate comparable results in different studies.

Taken together, we find evidence that Nakalanga syndrome and Nodding syndrome are closely related and may even be two manifestations of one underlying, yet unclear, pathology. According to the metaphoric hypothesis formulated by Wamala et al. [71] that “Nodding syndrome may be only the ears of the hippo,” Nakalanga syndrome may emerge to be one of these ears, i.e., the other end of a disease continuum. However, we want to caution against adoption of the premature conclusion that onchocerciasis is confirmed as the cause of Nodding—or Nakalanga—syndrome [72]. A multidisciplinary approach of researchers from various fields will be needed to find answers to the many unsolved questions connected with this (these) mysterious brain disorder(s).

**Key Learning Points**

- Nakalanga and Nodding syndromes both are developmental disorders of unknown cause affecting children and adolescents in onchocerciasis endemic areas of sub-Saharan Africa.
- Predominant features of Nakalanga syndrome are stunted growth, delayed pubertal development, and mental impairment—and epileptic seizures in a proportion of cases.
- Nodding syndrome is characterized by paroxysmal head nodding, frequently accompanied by mental decline—and stunted growth, delayed puberty, and convulsive seizures in a proportion of cases.
- Our review provides evidence that both syndromes are closely related and may represent manifestations of one underlying disease.
- We propose the use of clearly described multidisciplinary methods to clarify the relationship between these enigmatic disorders and their underlying pathology.

**Top Five Papers**

1. Raper AB, Ladkin RG. Endemic dwarfism in Uganda. East Afr Med J. 1950;27: 339–359.
2. Kipp W, Burnham G, Bamuhiiga J, Leichsenring M. The Nakalanga syndrome in Kabarole District, Western Uganda. Am J Trop Med Hyg 1996;54: 80–83.
3. Winkler AS, Friedrich K, König R, Meindl M, Helbok R, Unterberger I, et al. The head nodding syndrome—clinical classification and possible causes. Epilepsia. 2008;49: 2008–2015.
4. Dowell SF, Sejvar JJ, Riek L, Vandemaele KA, Lamunu M, Kuesel AC, et al. Nodding syndrome. Emerg Infect Dis. 2013;19:1374–1384.
5. Aall-Jilek LM. Epilepsy in the Wapagoro tribe in Tanganyika. Acta Psych Scand. 1965;41: 57–86.

## Supporting Information

S1 Diagram (PDF)

S2 Diagram (PDF)
