# Supplementary material for: Nakalanga Syndrome: Clinical Characteristics, Potential Causes, and Its Relationship with Recently Described Nodding Syndrome
Source: PLoS Negl Trop Dis. 2017 Feb 9;11(2):e0005201. doi: 10.1371/journal.pntd.0005201 (PMC5300103; doi:10.1371/journal.pntd.0005201)

**Nakalanga syndrome: Clinical characteristics, potential causes and its relationship with the recently described Nodding syndrome**

## Supplementary Information 1 (Diagram SI 1)

### Literature search for Nakalanga syndrome

**Objective:** To identify publications (case reports and case series) reporting original clinical information of patients affected by Nakalanga syndrome.
**Sources:** Medical Databases (Medline; ScienceDirect; African Neurology Database, Institute of Tropical Neurology, Limoges). Other sources: www-search with no defined limits, reference list of published articles.
**Procedure:** Search period: All years until present date, latest search May 23, 2016. Database search and screening for redundant entries performed by KF and CK. Search of other sources by KF, GG, EO, CK and AW. Verification of retrieved records for eligibility by KF and CK. (Criterion: Original data on clinical symptoms and signs of Nakalanga patients).
**Result:** 9 publications identified.

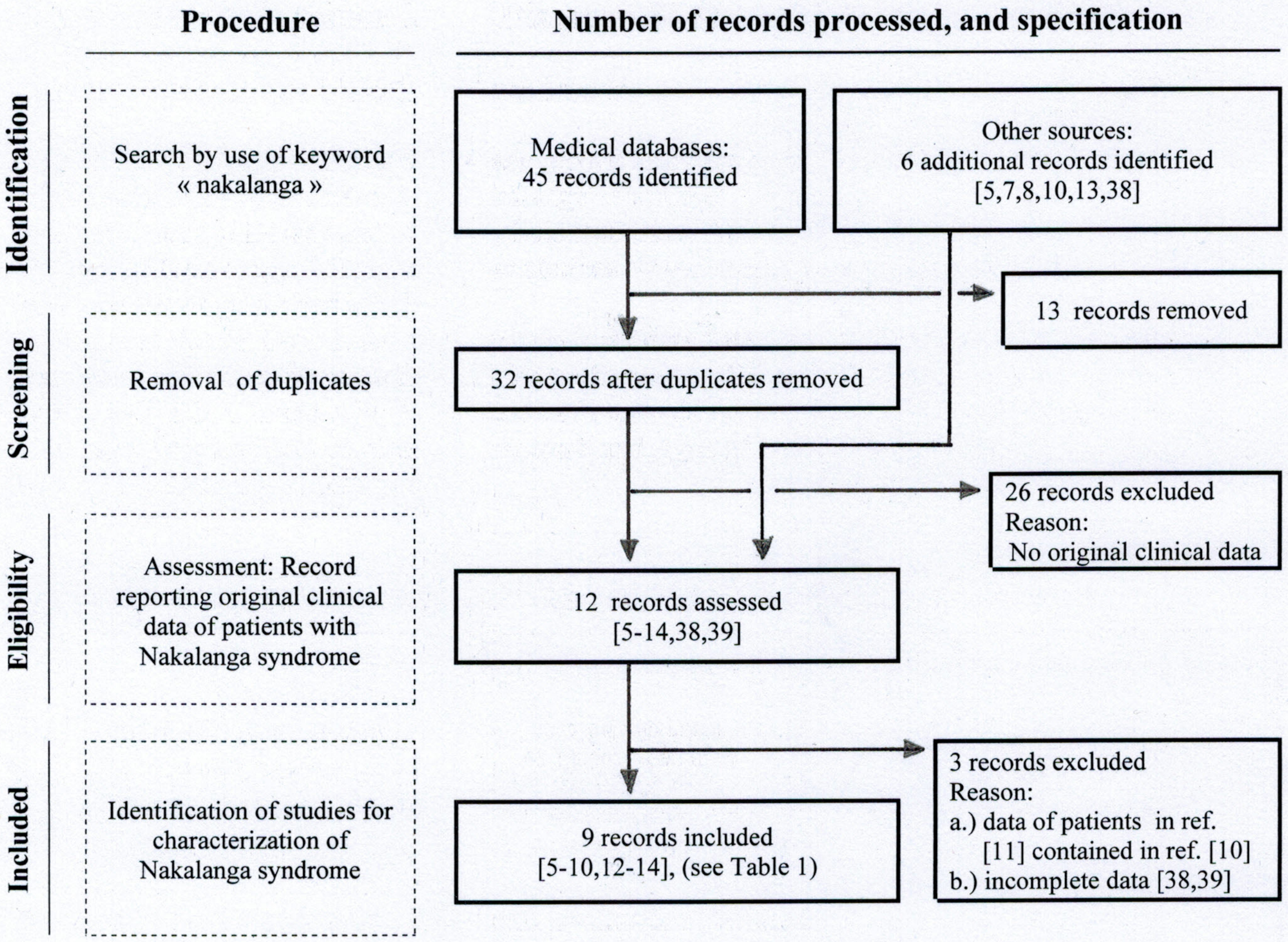

Supplement: S1 Diagram — (PDF) [file pntd.0005201.s001.pdf]
